# The Gothenburg H70 Birth cohort study 2014–16: design, methods and study population

**DOI:** 10.1007/s10654-018-0459-8

**Published:** 2018-11-13

**Authors:** Therese Rydberg Sterner, Felicia Ahlner, Kaj Blennow, Synneve Dahlin-Ivanoff, Hanna Falk, Lena Havstam Johansson, Maria Hoff, Mathias Holm, Helena Hörder, Tina Jacobsson, Boo Johansson, Lena Johansson, Jürgen Kern, Silke Kern, Alejandra Machado, Madeleine Mellqvist Fässberg, Johan Nilsson, Mats Ribbe, Elisabet Rothenberg, Lina Rydén, André Sadeghi, Simona Sacuiu, Jessica Samuelsson, Robert Sigström, Johan Skoog, Valgeir Thorvaldsson, Margda Waern, Eric Westman, Hanna Wetterberg, Henrik Zetterberg, Madeleine Zetterberg, Anna Zettergren, Svante Östling, Ingmar Skoog

**Affiliations:** 1 Centre for Ageing and Health (AgeCap) at the University of Gothenburg,; 2 Neuropsychiatric Epidemiology Unit, Department of Psychiatry and Neurochemistry, Institute of Neuroscience and Physiology, Sahlgrenska Academy at the University of Gothenburg, Gothenburg, Sweden; 3 Department of Clinical Neuroscience/Ophtalmology, Institute of Neuroscience and Physiology, Sahlgrenska Academy at the University of Gothenburg, Gothenburg, Sweden; 4 Unit of Audiology, Institute of Neuroscience and Physiology, Sahlgrenska Academy at the University of Gothenburg, Gothenburg, Sweden; 5 Division of Clinical Geriatrics, Department of Neurobiology, Care Sciences and Society, Karolinska Institutet, Stockholm, Sweden; 6 Department of Occupational and Environmental Medicine, Sahlgrenska University Hospital at the University of Gothenburg, Gothenburg, Sweden; 7 Department of Psychology, University of Gothenburg, Gothenburg, Sweden; 8 School of Education and Environment, Kristianstad University, Kristianstad, Sweden; 9 Institute of Neuroscience and Physiology, Department of Psychiatry and Neurochemistry, Sahlgrenska Academy at the University of Gothenburg, Mölndal, Sweden; 10 Clinical Neurochemistry Laboratory, Sahlgrenska University Hospital, Mölndal, Sweden; 11 Department of Molecular Neuroscience, University College London Institute of Neurology, London, UK; 12 UK Dementia Research Institute at UCL, London, UK; 13 Department of Health and Rehabilitation at Institute of Neuroscience and Physiology, Institute of Neuroscience and Physiology, Sahlgrenska Academy at the University of Gothenburg, Gothenburg, Sweden; 14 Department of Clinical Neuroscience, Institute of Neuroscience and Physiology, Sahlgrenska Academy at the University of Gothenburg, Gothenburg, Sweden

**Keywords:** H70 study, Ageing, Birth cohort, Population sample, Health, Study design

## Abstract

To improve health care for older persons, we need to learn more about ageing, e.g. identify protective factors and early markers for diseases. The Gothenburg H70 Birth Cohort Studies (the H70 studies) are multidisciplinary epidemiological studies examining representative birth cohorts of older populations in Gothenburg, Sweden. So far, six birth cohorts of 70-year-olds have been examined over time, and examinations have been virtually identical between studies. This paper describes the study procedures for the baseline examination of the *Birth cohort 1944,* conducted in 2014–16. In this study, all men and women born 1944 on specific dates, and registered as residents in Gothenburg, were eligible for participation (n = 1839). A total of 1203 (response rate 72.2%; 559 men and 644 women; mean age 70.5 years) agreed to participate in the study. The study comprised sampling of blood and cerebrospinal fluid, psychiatric, cognitive, and physical health examinations, examinations of genetics and family history, use of medications, social factors, functional ability and disability, physical fitness and activity, body composition, lung function, audiological and ophthalmological examinations, diet, brain imaging, as well as a close informant interview, and qualitative studies. As in previous examinations, data collection serves as a basis for future longitudinal follow-up examinations. The research gained from the H70 studies has clinical relevance in relation to prevention, early diagnosis, clinical course, experience of illness, understanding pathogenesis and prognosis. Results will increase our understanding of ageing and inform service development, which may lead to enhanced quality of care for older persons.

**Electronic supplementary material:**

The online version of this article (10.1007/s10654-018-0459-8) contains supplementary material, which is available to authorized users.

## Introduction

The number of people aged 60 years and above is globally expected to increase from 962 million in 2017 to 2.1 billion in 2050 [1]. Mental, cognitive and somatic health are major determinants of well-being in old age. To improve health care for older persons, we need to learn more about ageing, e.g. identify protective factors and early markers for diseases such as preclinical Alzheimer’s disease. To achieve this, it is necessary to conduct comprehensive studies on representative samples of older populations who are followed longitudinally.

**Fig. 1:**
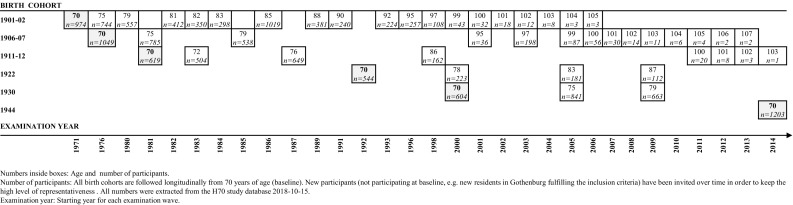
Overview of birth cohorts (age inside square) and examination years in the Gothenburg H70 Birth Cohort Studies

The Gothenburg H70 Birth Cohort Studies (H70 studies) are multidisciplinary epidemiological studies examining representative birth cohorts of older populations in Gothenburg, Sweden. The first study started in 1971. So far, six birth cohorts with baseline examination at age 70 have been followed longitudinally (see Fig. 1).

Study procedures for birth cohorts 1901–02, 1906–07 and 1911–12 have been described elsewhere [2, 3]. More than 700 papers have been published since 1971 (e.g. [4–13]). Examinations have been virtually identical between studies to enhance possibilities of comparisons between birth cohorts and examination years. This generates an opportunity to study time trends in age-related risk and protective factors, preclinical markers, as well as prevalence and incidence of diseases. In addition, new and modern types of assessments have been added.

The overarching aim of the H70 studies is to examine the impact of mental, somatic and social health on the functional ability and well-being of individuals aged 70 years and above, taking into account the complex interactions with age, sex, gender, socioeconomic gradients, environmental exposures, psychosocial, neurobiological, and genetic factors.

**Fig. 2:**
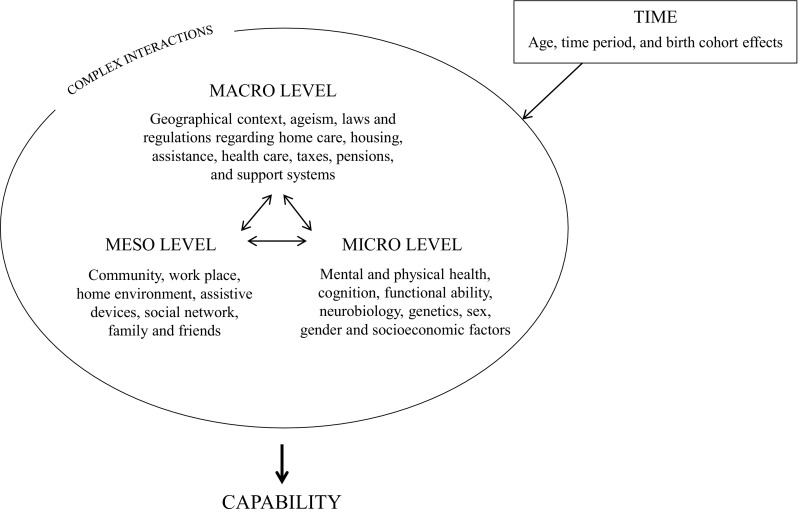
The underlying theoretical framework in the Gothenburg H70 Birth Cohort Studies

While there is no single definition of ageing, the phenomenon is often defined as an age associated decline of physiological and cognitive functions, and de-tuning of adaptive responses, followed by an increase in age-specific mortality [14]. Ageing is also related to positive dimensions, such as increase in wisdom and life experience. An underlying theoretical framework of the multidisciplinary H70 studies is the capability approach [15], aiming to illustrate contextual conditions of available resources for a specific person or population (Fig. 2).

This paper describes the study procedures for the baseline examination of the *Birth cohort 1944,* conducted in 2014–16. This was the largest and most comprehensive H70 study conducted so far. As in previous examinations, data collection serves as a basis for future longitudinal follow-up examinations.

## Methods

### Sample

Table 1 demonstrates the eligible, effective and total study samples. In line with previous studies in the H70 series, study participants were selected based on specific birth dates. Information regarding date of birth and residential addresses were obtained from the Swedish Tax Agency’s population register, which covers all persons registered as living in Sweden. In this study, all men and women born 1944 on dates ending with 0, 2, 5, or 8, and registered as residents in Gothenburg were eligible for participation (n = 1839). Persons were considered eligible irrespective of place of living (e.g. private households, sheltered living). Among those eligible, 29 individuals died before the examination, 32 had moved from Gothenburg, 53 could not speak Swedish, and 58 could not be traced, leaving an effective sample of 1667 individuals who were invited to participate in the study. The invitation was sent by letter, and included study information and a consent form. After approximately one week, a research staff member contacted the potential participants by telephone with an inquiry about study participation. In cases of no contact, up to three reminders were sent. A total of 1203 (response rate 72.2%; 559 men and 644 women; mean age 70.5 years) agreed to participate in the study. Table 2 shows demographic factors for all included participants.

**Table 1:** The H70 study sample (participants and non-participants) in 2014–16

	Women	Men	Total
Eligible sample^a^	961	878	1839
Excluded from sample	67	105	172
Could not be traced	26	32	58
Unable to communicate in Swedish	24	29	53
Not living in Gothenburg	9	23	32
Deceased	8	21	29
Effective sample	894	773	1667
Non-participants^b^	250	214	464
No given reason	108	87	195
Reported illness or injury	78	61	139
Regularly health check-ups	66	54	120
Relative declined	13	20	33
Too busy	14	20	34
Fear	18	14	32
Too healthy	8	5	13
Total sample	644	559	1203

^a^Residents in Gothenburg (including parts of the municipalities of Ale, Kungsbacka, Kungälv, Lerum, Mölndal) according to the Swedish Population Register, Swedish Tax Agency 2014

^b^Non-participants can be included in more than one self-reported category (one person wished to have their data destroyed after participation)

**Table 2:** Sample characteristics of 70-year-olds born 1944, participating in the H70 study 2014–16

	n	Men	Women	Total
Marital status, % (n)				
Having partner^a^	1196	80.5 (449)	62.1 (396)	70.7 (845)
Widowed	1196	4.3 (24)	11.9 (76)	8.4 (100)
Divorced	1196	10.4 (58)	22.9 (146)	17.1 (204)
Single^b^	1196	4.1 (23)	3.1 (20)	3.6 (43)
Other	1196	0.7 (4)	0 (0)	0.3 (4)
Education, % (n)				
Primary education ≤ 9 year	1192	19.3 (107)	15.2 (97)	17.1 (204)
More than primary education > 9 year	1192	80.7 (447)	84.8 (541)	82.9 (988)
University degree	1132	31.9 (169)	25.5 (154)	28.5 (323)
Country of birth, % (n)				
Sweden	1195	82.2 (458)	86.5 (552)	84.5 (1010)
Nordic countries	1195	5.2 (29)	5.3 (34)	5.3 (63)
European countries	1195	7.5 (42)	6.0 (38)	6.7 (80)
Other	1195	5.0 (28)	2.2 (14)	3.5 (42)
Average net income (per month), mean				
Individual income^c^	949	19,862	13,718	16,618
Individual pension^d^	740	17,783	13,047	15,109
Household income^c^	912	31,974	24,082	27,880
Average net income (per month), median				
Individual income^c^	949	16,500	12,000	14,000
Individual pension^d^	740	15,000	12,000	13,225
Household income^c^	912	28,900	22,000	25,000
Paid labour, % (n)				
Working part-time	1195	15.4 (86)	9.9 (63)	12.5 (149)
Working full-time	1195	4.8 (27)	0.6 (4)	2.6 (31)
Working in periods	1195	8.1 (45)	5.2 (33)	6.5 (78)
Housing, % (n)				
Sheltered living	1188	2.3 (13)	1.9 (12)	2.1 (25)
Mini-Mental State Examination, mean				
MMSE score^e^	1187	28.6	28.9	28.8
Smoking, % (n)				
Current smoker	1191	7.4 (41)	11.2 (71)	9.4 (112)
Previous smoker	1191	55.9 (310)	50.0 (318)	52.8 (628)
Having children, % (n)				
Yes^f^	1185	87.5 (484)	87.0 (550)	87.3 (1034)
Internet, % (n)				
Using internet every day	1137	68.5 (358)	63.5 (390)	65.8 (748)
Never using internet	1137	12.6 (66)	11.2 (69)	11.9 (135)

^a^Including living with partner, living apart from partner, and married

^b^Including single and never married/living with partner

^c^Including paid labour and pensions (SEK)

^d^Including only those not working and having pension as only income (SEK)

^e^MMSE has a maximum score of 30

^f^Including live biological and non-biological children

### Examinations

**Fig. 3:**
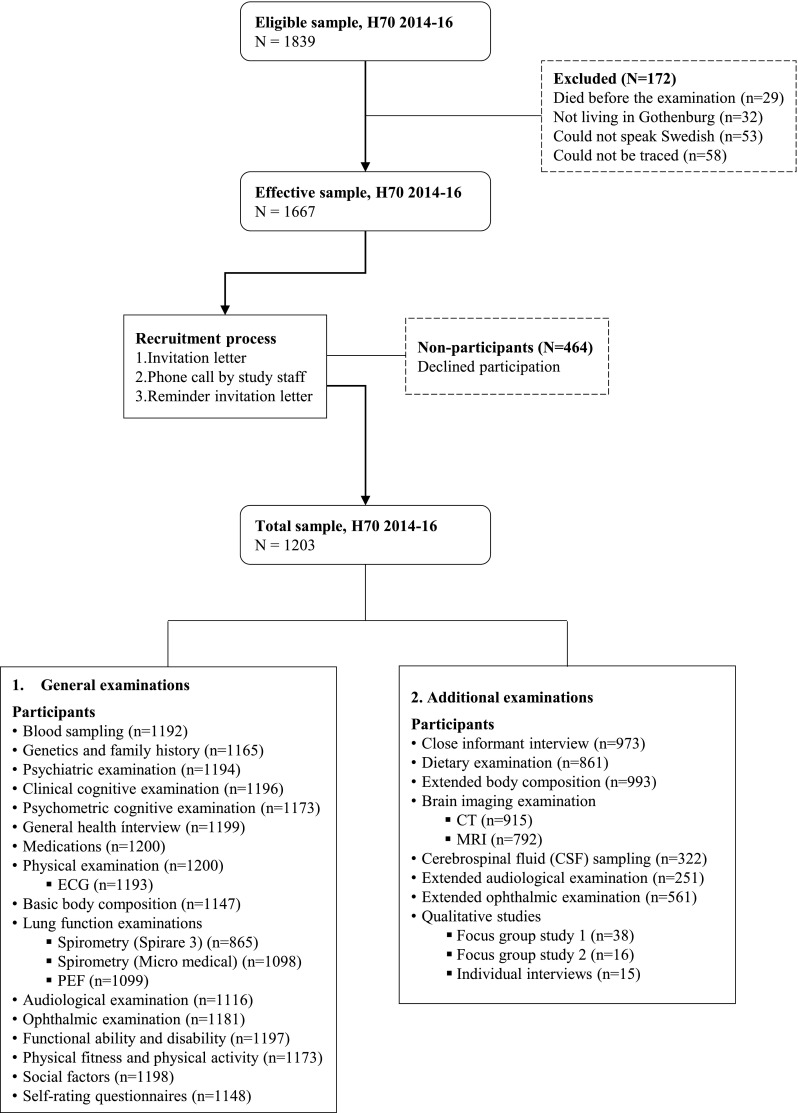
Sample flow chart for the H70 study 2014–16

**Fig. 4:**
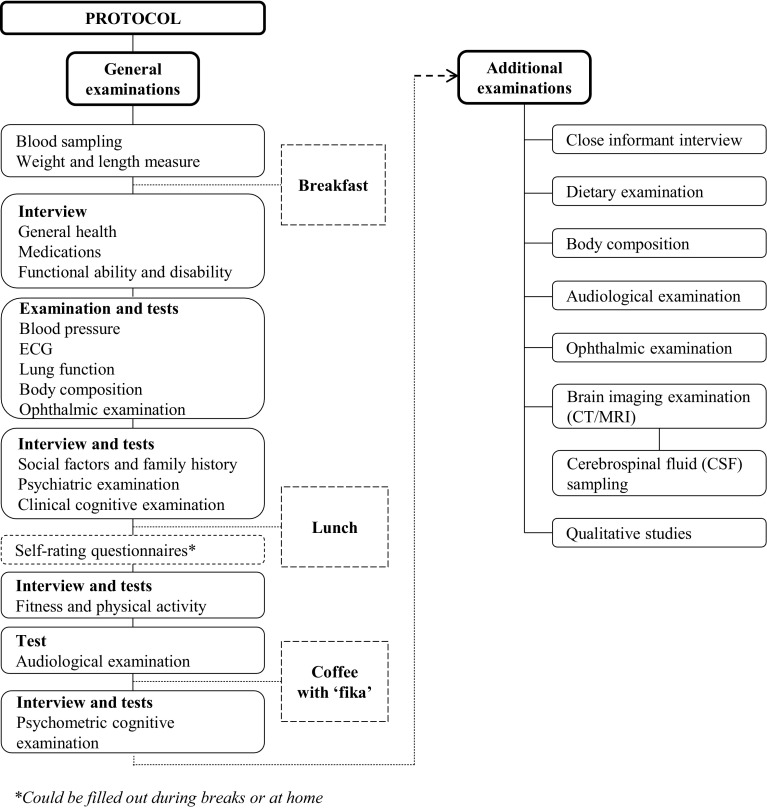
Study protocol for the H70 study 2014–16

The study comprised a one-day general examination at the Neuropsychiatric Clinic at Sahlgrenska University Hospital, as well as a number of additional examinations (see below). A sample flow chart is illustrated in Fig. 3, and a study protocol is illustrated in Fig. 4.

### General examinations

The general examinations were conducted at the Neuropsychiatric outpatient department at the Sahlgrenska University Hospital and lasted for approximately 8 h (including breakfast and lunch). Alternatively, the study participants could choose to divide the examination between 2 days, or choose to be examined only during home visits (n = 46). All interviews and tests were conducted by trained research staff in the H70 study team. All examinations are presented below.

#### Blood sampling

Blood sampling was performed by a research nurse in 1192 individuals (555 men, 637 women; response rate 99.1%) after overnight fasting. Fasting status was confirmed by the nurse before sampling. The venipuncture was performed in the inner elbow while the participant was in an upright sitting position. Blood sampling could not be performed in 11 individuals (due to e.g. small or deep veins). A maximum of 120 ml blood was drawn, of which 15 ml was used for blood tests. A maximum of 105 ml was aliquoted into serum (after 20–30 min of coagulation, 5 ml SST tubes were centrifuged with 2000 RCF (Relative Centrifugal Force) for 10 min and then pipetted into 1.5 ml micro tubes), plasma (10 ml EDTA tubes were centrifuged with 2000 RCF (Relative Centrifugal Force) in 20 °C for 15 min and then pipetted into 1.5 ml micro tubes) and whole blood (10 ml EDTA tubes pipetted into 1.5 ml micro tubes) and were frozen at –80 °C within approximately one hour to be saved in a biobank for future analyses according to the Swedish Biobank Law (2002:297) (biobank registration date: 2005-03-17; Registration Number: 532 at the National Board of Health and Welfare). All study participants gave written consent regarding biobanking. Blood tests included thrombocytes, hemoglobin (Hb), glucose, thyroid function (TSH, T4), kidney function (creatinine), liver function (AST, ALT), lipids (total-, HDL- and LDL-cholesterol, triglycerides) and indirect measurement of vitamin B12 and folic acid status (homocysteine). Name of the assay/reagent and instrument manufacturer for each measure can be found in Supplementary 1.

#### Genetics and family history

Due to a limited amount of blood sampled, DNA extraction was impossible to perform for 25 of the total 1192 individuals with blood samples. DNA was thus extracted from 1165 individual samples of whole blood (548 men, 617 women; response rate 97.7%). The extraction of DNA from whole blood was performed according to standard procedures at LGC Genomics in Berlin (Germany). All the DNA samples have been genotyped at University College London (UK), using *the Neuro Consortium Array (neurox2)* from Illumina (www.illumina.com). Additional single nucleotide polymorphisms (SNPs) in genes of specific interest have been, and will be, analyzed at LGC Genomics in Hoddesdon (UK), using the *KASP genotyping technology*. The participants and a close informant were asked about family history of dementia, depression, stroke, diabetes mellitus and cardiovascular disorders, as described below.

#### Psychiatric examination

A psychiatric examination was performed in 1194 individuals (555 men, 639 women; response rate 99.3%). Non-participants (n = 9) did not perform the examination due to several reasons (e.g. declining participation, aphasia or not speaking Swedish well enough to respond to the psychiatric questions). The semi-structured interview was conducted by a psychiatric research nurse, a psychiatrist, or a medical doctor, with a mean duration of 30 min. Psychiatric signs and symptoms during the past month were rated according to *the Comprehensive Psychopathological Rating Scale* (CPRS) [16], and *the Mini*-*International Neuropsychiatric Interview* (MINI) [17]. *The Montgomery*-*Åsberg Depression Rating Scale* (MADRS) [18] was derived from the CPRS to assess the overall burden of depressive symptoms. Both CPRS and MADRS have been found to be valid and reliable in older populations [19, 20]. The CPRS subscale, *The Brief Scale for Anxiety* (BSA) [21], was used to assess overall burden of anxiety symptoms. Mental and social functioning was assessed with the *Global Assessment of Functioning* (GAF) scale [22]. Questions about suicidal feelings were asked according to *Paykel* et al. [23]. The interview also comprised questions regarding symptoms of ADHD during early life and childhood [24], history of previous mental disorders; periods of hypomania according to Angst [25], the *Yale*-*Brown Obsessive*–*Compulsive Scale* [26], symptoms of depression and anxiety (panic, general anxiety, agoraphobia), specific phobias, social anxiety disorder, post-traumatic stress syndrome, psychotic symptoms, thoughts about death and suicide, feelings of loneliness, periods of stress in relation to family and work, cognitive symptoms, appetite and weight loss, and sleep. The examiner also rated global severity of mental health problems and a number of observed psychiatric symptoms. All diagnoses for psychiatric disorders (e.g. depression, generalized anxiety, specific and social phobia, bipolar disorder, psychosis, obsessive–compulsive disorder, and traumatic stress disorder) followed *the Diagnostic and Statistical Manual of Mental Disorders*, DSM-IV [27], DSM-IV-TR [28], and DSM-5 [29] criteria as closely as possible. Questions regarding mental health were also asked in self-rating questionnaires (see Table 3: GAF; Phobias; PSS-14).

#### Cognitive battery

A clinical cognitive examination was performed in 1196 individuals (557 men, 639 women; response rate 99.4%). The examination was conducted by a psychiatric nurse, a psychiatrist, or a medical doctor, with a total duration of approximately 25 min. The examination comprised tests of word fluency (animals), naming 12 objects, copying five figures, finger agnosia, orientation, selective attention, understanding proverbs, ideational apraxia, memory (remembering 12 objects, word memory list, two last prime ministers), ability to follow instructions. Cognitive function was rated in accordance with a Swedish version of *the Mini*-*Mental State Examination* (MMSE) [30], *the Gottfries Bråne Steen*-*scale* (GBS) [31], as well as global ratings. Specific assessments relevant for dementia diagnosis, such as recent and remote memory, semantic memory, concentration, apraxia, spoken language, language comprehension, word finding difficulties, and other intellectual functions were performed, as well as assessments with *the Clinical Dementia Rating scale* (CDR) [32], and *Alzheimer’s Disease Assessment Scale*-*Cognitive* (ADAS-COG) [33]. Dementia was diagnosed following the Diagnostic and Statistical Manual of Mental Disorders DSM III-R [34] criteria as closely as possible using information from the clinical cognition examination and information from key informants (see below). We also assessed the neurocognitive domains described in DSM-5 (complex attention, executive function, learning and memory, language, perceptual-motor, and social cognition) [29].

A more detailed psychometric cognitive examination that included questions about subjective memory as well as testing of multiple cognitive domains was performed in 1173 individuals (547 men, 626 women; response rate 97.5%). The examination was conducted by research staff members, all trained by a psychologist, with a total duration of approximately 60 min. The subjective part included questions concerning participant’s everyday memory, experiences of memory decline, satisfaction with own memory, everyday memory problems, memory training, and engagement in cognitively demanding recreational activities, such as chess, bridge, crossword-puzzles, and reading habits. The cognitive test battery included several cognitive measurements intended to cover a broad range of different cognitive abilities. *Memory in Reality* (MIR) [35], *Supra*-*span memory test* (BUS II) [36], and *Thurstone’s Picture Memory* [37] measured memory abilities. *Digit Span Forward and Backward test* [38] measured short term memory and executive functioning. Figure Identification—*Psif* [39] measured mental speed, and Figure Logic (*SRB 2*) [39] measured inductive reasoning. *Controlled Oral Word Association*—*FAS* (COWA-FAS) [40] measured verbal fluency and Block Design (*Koh’s Block Test*) [38] measured spatial ability. *The Clock Test* [41] was originally developed for dementia screening and measured a wide range of cognitive abilities including executive functioning [42]. Most of the tests are part of the *Dureman and Sälde* (1959) psychometric test battery [43] that was widely used in Sweden at the start of the H70 study 1971–72.

#### General health interview

A general health interview was performed in 1199 individuals (558 men, 641 women; response rate 99.7%), with a total duration of approximately 30 min. The interview was conducted by a research nurse or medical doctor, and comprised questions about health care, self-rated health, pain and fatigue, weight at age 20 and 50, current and past health issues (e.g. stroke/TIA, cancer, and a number of cardiovascular, respiratory, metabolic, endocrine, neurological, musculoskeletal, gastrointestinal, kidney, genitourinary, blood, eye and ear diseases) and treatment history. It also included questions about specific symptoms, e.g. chest pain according to the *Rose angina questionnaire* [44], breathlessness, dizziness/vertigo, headache, joint and low-back pain, falls and fractures, head trauma, dental health, hearing and eye sight, urinary and fecal incontinence, sexual behavior, age of menarche and menopause, hormonal and contraceptives use, and alcohol, drug and tobacco use.

Questions regarding self-rated health, substance use, and factors associated with physical health were also asked in self-rating questionnaires (see Table 3: SF-36, AUDIT and DUDIT).

#### Medications

A total of 1200 individuals (559 men, 641 women; response rate 99.8%) reported their medications, including dosages and treatment indications. Participants were also asked to bring their medication lists. The medications were classified according to the *Anatomical Therapeutic Chemical (ATC) classification system* recommended by the WHO [45].

#### Physical examination

Physical examinations were carried out by research nurses in 1200 individuals (559 men, 641 women; response rate 99.8%), with a total duration of approximately 45 min. The examination was conducted by a research nurse, and comprised measures of length, weight, waist and hip circumference, ankle pressure (Minidop ES-100VX Hadeco), and pulse frequency. Systolic and diastolic blood pressure were recorded in the right arm in the sitting position after 5 min rest using a standard cuff (Umedico). Blood pressure was also recorded in the supine position after 5 min rest, and after one and 3 min in the standing position. The neurological examination included the *Romberg test* [46], the *Grasset test*, and evaluation of hypokinesia, rigidity, tremor, and a number of other motor disturbances. Electrocardiography (ECG) was performed in 1193 individuals (556 men, 637 women; response rate 99.2%) (SCHILLER AT-2 plus or SCHILLER AT-102 plus at the outpatient clinic, and SHILLER AT-101 during home visits). Non-participants (n = 10) did not perform the examination due to several reasons (e.g. declining participation or technical reasons). The ECG was coded according to *the Minnesota code system* [47].

#### Body composition examination

Bioelectrical impedance was performed in 1147 individuals (533 men, 614 women; response rate 95.3%). Non-participants (n = 56) did not perform the examination due to several reasons (e.g. having pace-maker or wheelchair). The examination was used to measure individual body composition (Body Composition Analyzer TANITA T7063 09010007 at the outpatient clinic, and TANITA T6360 07060054 during home visits). Participants were also asked to take part in a more comprehensive body composition examination (see below).

#### Lung function

A total of 865 individuals (412 men, 453 women; response rate 71.9%) performed a lung function examination. This comprised spirometry (Spirare SPS310 sensor and Spirare 3 software, Diagnostica AS, Oslo, Norway) without bronchodilator administration according to *the American Thoracic Society/European Respiratory Society standards* [48]. Maximum forced volume capacity (FVC), forced expiratory volume in 1 s (FEV1), peak expiratory flow (PEF), forced expiratory flow (FEF25, 50, 75), maximal (mid-) expiratory flow (MMEF), slow vital capacity (SVC), and forced expiratory time (FET) were measured.

As in previous H70 studies, FVC and FEV1 were also measured with a Micro Medical portable spirometer (Micro Medical Ltd, Rochester, Kent, UK) [49], and peak expiratory flow (PEF) was recorded with a Mini-Wright Peak Flow Meter (Clement Clarke International Ltd, Harlow, Essex, UK) [50], where participants were asked to exhale with a maximally forced effort from a position of maximal inspiration. A total of 1098 (515 men, 583 women; response rate 91.3%) individuals performed the Micro Medical portable spirometer test (FVC, FEV1), and 1099 (518 men, 581 women; response rate 91.4%) performed the Mini-Wright Peak Flow Meter test (PEF). Each participant performed the PEF test three times and the highest value was used as the final result. Of those who did not perform the lung function examinations, several reasons were reported (e.g. technical reasons or physically unable to take the test). Only the Micro Medical portable spirometer and Mini-Wright Peak Flow Meter could be performed during home visits.

#### Audiological examination

An audiological examination was performed in 1116 individuals (523 men, 593 women; response rate 92.8%), with a total duration of approximately 30 min. Non-participants (n = 87) did not perform the examination due to several reasons (e.g. declining participation or technical reasons). The examination could not be performed during home visits. The audiological examination was conducted by a research nurse in a quiet office setting, and comprised pure tone audiometry, which measures the sensitivity of the ear to tones of differing frequency; wide band tympanometry, measuring the condition of the middle ear and the mobility of the ear drum and the construction bones; and otoscopy. Audiometry (air-conduction only) was carried out with an automated method using Sennheiser HDA200 headphones. The examination maps hearing loss caused by lesions and diseases in the middle ear and the cochlear nerve. This type of audiometry is gold standard for clinical practice and for epidemiological purposes. Questions were also asked about perceived hearing disability, hearing aid use and tinnitus. A subsample performed a more comprehensive audiological examination (see below).

#### Ophthalmic examination

Near vision acuity was tested with *a Jaeger eye chart* in 1181 individuals (549 men, 632 women; response rate 98.2%) with the participants’ own reading glasses if any. The general examination also included questions about ocular morbidity and vision. Questions regarding visual-related quality of life were also asked in self-rating questionnaires (see Table 3: VFQ-25). A subsample performed a more comprehensive ophthalmic examination (see below).

#### Functional ability and disability

An interview assessing functional ability and disability was performed in 1197 individuals (558 men, 639 women; response rate 99.5%). The interview was conducted by a research nurse, with a total duration of approximately 20 min. Difficulty in self-care tasks or activities of daily living (ADL) was either self-assessed, based on observations by the research nurse, or from information from staff and carers. Several instruments were used. The *Barthel Index of Activities of Daily Living* [51–53] included ten domains of function (bowels, bladder, grooming, toilet use, feeding, transfer, mobility, dressing, stairs, and bathing). Response options were either independent or dependent/unable. A summary score ranged from zero (low function, dependent) to 100 (high function, independent). The *Katz Index of Independence in ADL* [54] included six domains of function (bathing, dressing, toileting, transferring from bed to chair, continence, and feeding), with response options independent or dependent. A summary score ranged from zero (high function/independent) to six (low function/dependent). Independent living skills or instrumental activities of daily living (IADL) were measured with the *Lawton Instrumental Activities of Daily Living Scale* (IADL) [55]. The scale provides information about functional skills necessary to live in the community, and includes eight domains of function (ability to use the telephone, shopping, food preparation, housekeeping, laundry, mode of transportation, responsibility of own medications, and the ability to handle finances). Response options ranged from fully independent to completely unable/incapable. A summary score ranged from zero (low function, dependent) to eight (high function, independent). The examination also included assessment of a number of other functions used in previous H70 studies. The “ADL staircase” included a mix of questions from the Katz and Lawton scales with response options independent/dependent. Questions were also asked about use of assistive devices, ability to pick up things from the floor, ability to move around indoors and outdoors with and without assistive devices, past year travel abroad, car driving, and use of community health care, services, and personal care needs including help from family and friends. Questions regarding helping others with daily activities were also asked in self-rating questionnaires (see Table 3: Relationships).

#### Physical fitness and physical activity examination

An examination of fitness and physical activity was performed in 1173 individuals (545 men, 628 women; response rate 97.5%), with a total duration of approximately 30 min. A physiotherapist performed the examination, which included fitness tests and an interview regarding physical activity. *Self*-*selected and maximum gait speed* [56] for 30-meter indoors with standing start were measured (meter/second), as well as achieved walking distance (meters) during a *six*-*minutes indoor walking test* [57]. *Pulse and oxygen uptake* were measured at four time points with a pulse oximeter (NONIN Onyx II model 9550 oximeter). *Grip strength* was tested with a *Martin Vigorimeter* [58] and *a JAMAR dynamometer* (sub-sample) [59], with the shoulder joint in a neutral position. The test was repeated three times for each hand, and the highest value of the best hand was used as outcome. *Timed chair*-*stand* [60] tested the ability to stand up and sit down from a chair (height 43 cm) five times in a row as quickly as possible (five-repetition sit-to-stand-test) [61]. The total time (in seconds) was used as outcome. *One*-*leg static balance* [62] tested the ability to stand on one leg without shoes, for a maximum of 30 s, with eyes open. The test was interrupted if the individual moved from the standardized position and three trials for each leg were allowed. A *dynamic balance test* [63, 64] was also performed, in which the study participants were instructed to walk along the lines of a floor-painted “8” two times. Both time (in seconds) and number of inaccurate steps (counting left and right) were registered. A *stair climbing test* was conducted, as described in [65], testing the ability to climb onto a box of 50 cm (or 40, 30, 20 to 10 cm, if unable to climb the box of 50 cm) with or without support using a handle attached to the wall. Physical activity was assessed by an interview including the 6-grade physical activity scale [66] and study specific questions regarding walking habits and types of physical activities. The examination also comprised questions about walking habits, self-rated fitness, level of physical activity at certain ages (lifetime), participation in competitive sports (lifetime), and type of activities performed during the past 12 months. Questions regarding physical activity were also asked in a self-rating questionnaire (see Table 3: IPAQ).

#### Social factors

An interview regarding social factors was performed in 1198 individuals (558 men, 640 women; response rate 99.6%), with a total duration of approximately 30 min. The interview was conducted by a research nurse, and comprised questions about past and present marital status and marital satisfaction, relationships with friends, partner’s physical and mental health, and need of assistance, housing and neighborhood, noise in surrounding, place of birth and family origin, family constellation and number of children, grandchildren, and siblings, and death of children, grandchildren, siblings or friends. Questions were also asked about serious illness and social problems in children and grandchildren, conflict with children or grandchildren and time spent helping them in everyday life, parents’ age at birth of participant, parents’ age at death, and own age at death of parent. Age at time of move to Gothenburg was recorded for those not born in the city. Study participants were also asked about childhood conditions, education, past and present working conditions, retirement and reaction to retirement transition, and partner’s work or retirement status, and current feelings of loneliness. Questions regarding social factors were also asked in self-rating questionnaires (see Table 3: Activities, Relationships, Income, and Work), which included questions about e.g. hobbies, leisure time activities, and use of internet and computers.

#### Self-rating questionnaires

All participants were asked to answer 19 self-rating questionnaires. Number of participants answering each questionnaire can be seen in Table 3. The self-rating questionnaires could be filled out during the day of the general examinations or at home. In the latter case, questionnaires were sent back by mail. A continuous quality assurance comprising completion of unanswered questions was made by the research staff by telephone and letter. A total of 47 participants (23 men, 24 women) declined to answer all questionnaires (due to cognitive decline, impaired vision, poor health, or unwillingness to answer).

**Table 3:** Overview of the self-rating questionnaires included in the H70 study 2014–16

Instrument	n (Men, women; response rate)	Content	References
Capability			
ICECAP-O	1040 (482 men, 558 women; 86.5%)	The ICEpop CAPability measure for Older people comprises five questions measuring individual quality of life with a capability approach	[84]
Health			
GAF	1130 (519 men, 611 women; 93.9%)	The Global Assessment of Functioning comprises two questions measuring the social, occupational, and psychological functioning	[22]
PHOBIAS	1134 (522 men, 612 women; 94.3%)	Specific phobia is measuring with 60 items (e.g. snakes, elevators, blood)	^a^
PSS-14	1135 (523 men, 612 women; 94.3%)	The Percieved Stress Scale comprises 14 questions measuring general perceived stress	[85]
SF-36	1137 (524 men, 613 women; 94.5%)	The Short-Form Health Survey comprises 36 questions measuring physical, mental and social health	[86]
SOC	1138 (525 men, 613 women; 94.6%)	The Sense of Coherence questionnaire comprises 13 questions measuring comprehensibility, manageability, and meaningfulness	[87]
VFQ-25	1139 (525 men, 614 women; 94.7%)	The Visual Function Questionnaire comprises 25 questions measuring global vison, difficulty with near and distance activities, limitations in social functioning, role limitations, dependency on others and mental health symptoms due to vision, driving difficulties and ocular pain	[88]
Life style			
ACTIVITIES	1140 (524 men, 616 women; 94.8%)	Leisure time activities and interests, religious belief, and media consumption are measured with 60 questions	^a^
AUDIT	1134 (520 men, 614 women; 94.3%)	The Alcohol Use Disorders Identification Test comprises 10 questions about alcohol consumption measuring frequency, amount (standard drinks), and alcohol-related dependence, consequences and harm	[89]
DIET	1142 (526 men, 616 women; 94.9%)	Dietary pattern (quantity and variety when consuming specific food products) is measured with 16 questions	^a^
DUDIT	1137 (524 men, 613 women; 94.5%)	The Drug Use Disorders Identification Test comprises 10 questions about drug consumption (e.g. cannabis, cocaine or overconsumption of prescribed medication) measuring frequency, amount and dependence, consequences and harm	[90]
EDI	1143 (528 men, 615 women; 95.0%)	Body image and emotions attitudes towards eating are measured with 13 questions	[91]
IPAQ	1040 (482 men, 558 women; 86.5%)	The International Physical Activity status Questionnaire comprises 10 questions measuring physical activity during the past 7 days	[92]
RELATIONSHIPS	1138 (525 men, 613 women; 94.6%)	Social network (family, friends, neighbors and pets) is measured with 39 questions	^a^
Personality			
CMPS^b^	242 (116 men, 126 women; 82.0%)	The Cesarec Marke Personality Scheme comprise 165 questions measuring the personality traits achievement, affiliation, aggression, defense of status, guilt feelings, dominance, exhibition, autonomy, nurturance, order and succorance	[93]
EPI	1140 (526 men, 614 women; 94.8%)	The Eysenck Personality Inventory comprises 57 questions measuring the personality traits extraversion and neuroticism	[94]
NEO-FFI-3	1141 (524 men, 617 women; 94.8%)	The NEO Five Factor Inventory comprises 60 questions measuring the personality traits neuroticism, extraversion, openness, agreeableness, and conscientiousness	[95]
PN-SRI	1138 (526 men, 612 women; 94.6%)	The Positive–Negative Sex-Role Inventory comprises 24 questions measuring gender coded personality traits within femininity, masculinity, and androgyny	[96, 97]
Socioeconomic status			
INCOME	1121 (518 men, 603 women; 93.2%)	Individual income and total household income are measured with six questions	^a^
WORK	1127 (518 men, 609 women; 93.7%)	Previous and current working states, together with ratings of subjective work capacity are measured with three questions	^a^

^a^Self-constructed questionnaire

^b^Only a subsample (n = 295) were asked to answer the *Cesarec Marke Personality Scheme* (CMPS), to increase comparability of personality traits measured in previous phases of the H70 study

#### Final rating of medical burden

Following the general examination, an overall quantitative rating of medical burden was made using *the Cumulative Illness Rating Scale for Geriatrics (CIRS*-*G)* [67, 68]. Medical problems were rated by organ system (heart, vascular, hematopoietic, respiratory, eyes, ears, nose, throat and larynx, upper gastrointestinal tract, lower gastrointestinal tract, liver, renal, genitourinary, musculoskeletal and skin, neurological, endocrine, metabolic and breast, and psychiatric illness) with a scale ranging from 0 to 4, from no problems to extremely severe.

### Additional examinations

After the general examination, all study participants were asked to take part in additional examinations at a later date: close informant interview, dietary examination, body composition examination (DXA, BIS), computed tomography (CT-scan) and magnetic resonance imaging (MRI) of the brain, and lumbar puncture (LP). Subsamples were invited to extended audiological, extended ophthalmological examinations, and qualitative studies. All additional examinations are presented below.

#### Close informant interview

All participants were asked if they would like to supply contact information for a close informant for a collateral interview by research staff. If the participant was not able to provide this information due to dementia, a close informant was sought in other ways (e.g. through staff at residential care facilities). A close informant interview was performed by telephone for 973 individuals (470 men, 503 women; response rate 80.9%). Among those not having a close informant interview (n = 230), several reasons were reported (e.g. did not have any close informants to contact, close informants declined to participate). The interview was conducted by a research nurse or psychologist, during a mean of 45 min. The close informant was a spouse or partner in 606 cases, a daughter in 176 cases, a son in 79 cases, and others (e.g. friend, grandchild, or residential care staff) in 53 cases. The mean interval between the general examination and the close informant interview was 209 days. The close informant interview was semi-structured, and included *the Informant Questionnaire on Cognitive Decline in the Elderly* (IQCODE) [69], which assesses changes in everyday cognitive function. The interview also comprised questions regarding cognitive symptoms and functional abilities (e.g. memory, spatial orientation, language, general intellectual symptoms, eating habits, incontinence, and a number of activities of daily living), as well as psychiatric symptoms (e.g. personality changes, irritability and aggressive behavior, motivation, emotions, depressive, anxiety, obsessive compulsive, psychotic and other psychiatric symptoms), and age at onset, course and cause of symptoms or disabilities. The interview also included questions regarding a number of background factors (e.g. education, smoking, snoring, family situation, and life events), history of diseases (e.g. cerebrovascular and cardiovascular diseases, dementia, head trauma, alcohol abuse, infectious diseases, deficiency states, hypo- and hyperthyroidism, chronic bronchitis and asthma, diabetes mellitus, Parkinson’s disease, normal pressure hydrocephalus, hip fracture), and family history of diseases (dementia, depression, stroke, cardiovascular diseases, diabetes mellitus). In cases of dementia, questions were also asked regarding course and age at onset.

#### Dietary examination

All study participants without dementia were invited to take part in a dietary examination. The examination was performed in 861 individuals (385 men, 476 women; response rate 71.6%) with a total duration of approximately 60–90 min. Non-participants (n = 342) did not perform the examination due to several reasons (e.g. declining participation, poor health, lack of time or no response). A dietician performed a semi-structured interview capturing habitual dietary intake (food and beverages) during the past 3 months. The interview, which was conducted at the outpatient clinic or during a home visit, included not only open-ended questions about usual food patterns, but also structured questions in order to capture total intake as closely as possible. Dietary intake, defined as energy and nutrient intake, and food pattern were measured by the diet history (DH) method described elsewhere [70, 71]. Data from DH interviews was processed using the Swedish National Food Agency’s nutrient database (The Swedish Food Composition Database), to estimate energy and nutrient intake.

#### Extended body composition (DXA, BIS)

All study participants were invited to take part in a body composition examination. The examination was performed in 993 individuals (402 men, 591 women; response rate 82.5%) with total duration of approximately 20 min. Non-participants (n = 210) did not perform the examination due to several reasons (e.g. declining participation, or technical reasons or no contact). The body composition examination was conducted by a nurse at Sahlgrenska University Hospital, where dual energy X-ray absorptiometry (DXA) was measured using a Lunar Prodigy scanner (Scanex, Helsingborg, Sweden) [72]. A full body scan analyzed the amount of body fat, lean soft tissue, and bone mineral content. Fat-free mass was defined as the sum of lean soft tissue and bone mineral content. Appendicular lean soft tissue was defined as the sum of lean soft tissue in arms and legs. Body composition was measured by bioelectrical impedance spectroscopy (BIS) using an ImpediMed SFB7 device. BIS measures body composition based on the principle that tissues in the body conduct electric current. BIS calculated total body water and the amount and distribution of intracellular water and extracellular water via electrodes on hand/wrist and foot/ankle. Thus, fat free mass was estimated. Resistance and reactance measurements were made over a wide range of frequencies [73, 74]. Manufacturer’s proprietary software was used to calculate Cole Plot parameters and body composition estimates.

#### Computed tomography (CT-scan) and magnetic resonance imaging (MRI)

All study participants were invited to take part in a brain imaging examination, conducted at the Aleris Clinic in Gothenburg. Participants underwent both computed tomography (CT, approximately 5 min) and magnetic resonance imaging (MRI, approximately 40 min) during the same session. Number of participants and the acquisition parameters for CT and MRI can be observed in Table 4. The CT examination was performed in 915 individuals (response rate 76.1%), and the MRI examination in 791 individuals (response rate 65.8%). Participants were interviewed for safety procedures (e.g. incompatible implant devices such as pacemakers) and informed consent was obtained. Non-participants did not perform the examination due to several reasons (e.g. declining participation, no response or expressing fear or discomfort). For those willing to undergo MRI, five could not participate due to contraindications (i.e. pace-makers). The examination was interrupted on 22 occasions due to claustrophobia and three occasions due to noise discomfort.

**Table 4:** Computed tomography (CT-scan) and Magnetic Resonance Imaging (MRI) acquisition parameters in the H70 study 2014–16

CT	n (men/women)	Slice thickness	Rot. time	Pitch	Increment	Acquisition matrix (mm)	Filter	Windowing
Brain helical	434/481	0,9	0.4	0.392	0.45	512 × 512	Brain std (UB)	C35 W70

Series description MRI	n (men/women)	Slice thickness	Repetition time (ms)	Echo time (ms)	Inversion time (ms)	Acquisition matrix (mm)	Field of view (mm)	Flip angle (°)
T1W_3D_TFE SENSE (Sag)	377/414	1	7.2	3,2	–	250 × 250	256 × 256	9
3D_FLAIR sag SENSE	376/414	2	4800	280	1650	250 × 237	250 × 250	90
T2w (tra)	376/413	4	3000	80	–	288 × 223	230 × 230	90
VEN_BOLD_HR (SWI)	376/413	1	14,59–17,60	20,59–24,99	–	220 × 222	220 × 220	10
rs-fMRI	365/396	4	2500	14	–	64 × 64	240 × 240	70
High-ISO DTI	365/394	3	7340	83	–	112 × 112	224 × 224	90

The CT data was acquired on a 64-slice Philips Ingenuity CT system (Philips Medical Systems, Best, Netherlands). The MRI data was acquired on a 3.0T Philips Achieva system (Philips Medical Systems). The MRI protocol consisted of a T1 3D-isotropic acquisition (T1W_3D_TFE) for structural changes, a fluid attenuation inversion recovery (FLAIR) for detection of white matter pathology, and T2 weighted images (t2w) to exclude other types of pathologies such as tumors. Further, a Venus bold sequence was acquired for the detection of microbleeds. White matter integrity was examined using diffusion tensor imaging (DTI) sequence and functional changes were observed with resting-state fMRI sequence.

#### Cerebrospinal fluid (CSF) sampling

All study participants were invited to take part in CFS sampling via a lumbar puncture (LP). A total of 430 individuals consented (response rate 35.7%), but there were pharmacological contraindications for 108 persons (e.g. anticoagulant therapy, immune modulation, cancer therapy), leaving 322 participants (166 men, 156 women). Of those unwilling to participate (n = 773), several reasons were reported (e.g. expressing fear or anticipated discomfort regarding the examination or its results). The LP, with total duration of approximately 30 min, was conducted by a consultant neurologist/psychiatrist at the Neuropsychiatric outpatient department at the Sahlgrenska University Hospital. Prior to the examination, each participant had to undergo CT and/or MRI examination in order to detect possible contradictions. All CSF samples were collected by LP in the morning from the L3/L4 or L4/L5 interspace. One CSF aliquot was used for CSF analyses [75], including a CSF cell count and the CSF/serum albumin ratio, after which a total of 10 ml of CSF was collected in a polypropylene tube and immediately transported to the local laboratory for centrifugation at 1800 RCF (Relative Centrifugal Force) at 20 °C for 10 min. The supernatant was gently mixed to avoid possible gradient effects, aliquoted in polypropylene tubes and stored at − 70 °C. CSF samples in the present study underwent one freeze–thaw cycle prior to analysis. CSF total-tau, phospho-tau and Aβ42 were analyzed directly after 1 to a few days at − 20 °C. CSF total tau and tau phosphorylated at threonine 181 (p-tau) were determined using a sandwich enzyme-linked immunosorbent assay (ELISA) (INNOTEST^®^ htau Ag and PHOSPHO_TAU (181P), (Fujirebio, Ghent Belgium) as previously described [76, 77]. CSF amyloid-β42 (Aβ42) peptide was measured using a sandwich ELISA (INNOTEST^®^ β-amyloid_1-42_), specifically constructed to measure amyloid-β peptides starting at amino acid 1 and ending at amino acid 42 [78]. All measurements were performed by board certified laboratory technicians blinded to clinical data. Laboratory procedures were accredited by the Swedish Board for Accreditation and Conformity Assessment. The laboratory technicians were blinded to clinical data.

#### Extended audiological examination (see also Supplementary 2)

A subsample of 305 participants, born on dates ending with 2 and 5 of each month, were invited for an extended audiological examination conducted at the Audiology Unit, Sahlgrenska Academy. Of those invited, 251 participants (113 men, 138 women; response rate 82.3%) underwent the examination with total duration of approximately 90 min.

#### Extended ophthalmic examination (see also Supplementary 3)

A subsample of 631 participants, born on days ending with 0, 2, 5 and 8 (born January-April), and 5 and 8 (born May-December), were invited for an ophthalmic examination conducted at Sahlgrenska University Hospital, Mölndal. Of those invited, 561 participants (266 men, 295 women; response rate 88.9%) underwent the examination with a total duration of approximately 90 min.

#### Qualitative studies (see also Supplementary 4)

Purposive sub-samples of study participants also took part in qualitative studies (focus groups or individual interviews).

#### Registry data (see also Supplementary 5)

The H70 study is permitted to be linked to a large number of databases from different state agencies in Sweden (e.g. including data regarding participants’ birth records, visits to early child care, schooling, drafting records for 18-year-old men).

### Ethical approval and informed consent

The H70 study was approved by the Regional Ethical Review Board in Gothenburg (Approval Numbers: 869-13, T076-14, T166-14, 976-13, 127-14, T936-15, 006-14, T703-14, 006-14, T201-17, T915-14, 959-15, T139-15), and by the Radiation Protection Committee (Approval Number: 13-64).

Study participants provided written informed consent prior to the general and additional examinations. Consent was obtained from a relative if the participant was unable to provide own consent. Travel checks for local transportation were offered upon request. Taxis were booked and pre-paid by the study for participants in need of assisted transportation. All participants examined at the Neuropsychiatric outpatient department were offered complimentary breakfast, lunch and afternoon snack. No other financial compensation was given.

Results on biomarkers (blood samples, ECG, blood pressure, CT/MRI scans and lumbar puncture) were communicated to the study participants by letter. With the approval of the participant, a copy was sent to the primary care facility. Abnormal test results, previously undetected disease and acute medical conditions were first handled by the responsible study physician, who initiated appropriate referrals. Upon detection of acute medical conditions, study participants were transported to the emergency service at the Sahlgrenska University Hospital in Gothenburg.

### Data management

Data are stored at the Institute of Neuroscience and Physiology at the University of Gothenburg according to GDPR regulations. A database group is responsible for quality control, data cleaning, and data delivery. Data will be made available to outside researchers through our standard security process, and according to established GDPR data sharing guidelines. Requests for data will be considered by the H70 study data sharing committee on the basis of scientific priorities and overlapping interests. Contact information can be found at www.epinep.gu.se. In line with the FAIR Principles, information about the studies, selected questionnaires and metadata are posted at the Swedish National Data Service (www.snd.gu.se). The database is also part of NEAR (National E-infrastructure for Aging Research in Sweden) financed by the Swedish Research Council.

## Discussion

The Gothenburg H70 Birth Cohort Studies comprise longitudinal examinations of older birth cohorts born between 1901 and 1944. The examination of 70-year-olds born 1944 was the largest and most comprehensive study conducted so far, comprising more than 1200 persons. The sample was highly educated (almost 30% had a university degree), and had high cognitive function (mean MMSE score 28.8). Current employment (at least part time) was reported by one-fifth. Almost 90% had children, 71% had a current partner, two-thirds used internet daily, and only 2% had sheltered living. Thus, it is a population sample with high cognitive and social functioning.

Researchers involved in the multidisciplinary studies include psychiatrists, molecular biologists, neurologists, geriatricians, audiologists, ophthalmologists, dieticians, rheumatologists, health care professionals, public health professionals, physiotherapists, occupational therapists, epidemiologists, geneticists, neuroradiologists, neurochemists, media researchers, historians, psychologists, sociologists and economists. The diverse nature of the research team reflects the complexity of the ageing process and the need for comprehensive studies examining relationships between various age-related factors and health related outcomes on micro, meso, and macro levels. Several challenges need to be addressed when conducting comprehensive longitudinal population based studies.

One challenge is participant exhaustion. The magnitude of the study may be perceived as demanding, especially for those having a high burden of disease. However, our experience from the H70 studies is that this is a minor problem. Most participants take part in most examinations, and participants in previous cohorts have returned to follow-up examinations over several decades generating high response rates. In addition, participants could opt to take the examination over several days, and home visits were also offered as an alternative.

Another challenge is to keep response rates as high as possible to secure representativeness. Worldwide, the willingness to participate in scientific studies has decreased dramatically during the last decades [82]. Previous H70 studies yielded declining response rates from 85% in 1971–72, to 81% in 1976–77, 77% in 1981–82, and 70% in 2000. This declining trend was broken in 2014–16 (response rate of 72%), which may be due to intensified and repeated recruitment efforts. A common reason for non-participation in 2014–16 was suffering from an illness or injury that made it difficult to participate. However, another common reason was the view that participating in the H70 study would not generate any added value as the individual already had regular check-ups in primary care, and expressed feeling in control over own health. In addition, a few expressed being too healthy and suggested that we instead should focus on persons in need of health care. Thus, reasons for declining included both being too ill and being too healthy. This may have generated a sample with diverse health status. Relatives/friends declined participation on behalf of the invited persons in 33 cases, sometimes also after that the individual had agreed to participate. Although we accepted this on all occasions, it can be questioned in cases in which the mental health of the potential participant is uncompromised. Another factor, which may hamper representativeness, is that participants needed to be able to speak Swedish, as we did not have resources to use translators. In addition, translators may not be optimal with the very sensitive questions asked in this study, e.g. the psychiatric interview. We will thus not be able to generalize our findings to persons who do not speak Swedish. However, the proportion born outside Sweden in our study (16%) is only slightly lower than that of the total same-age population in Gothenburg (19%, Statistics Sweden).

A third challenge with epidemiological studies conducted over several decades, by several generations of researchers, is to keep measurements and assessments as consistent as possible to enhance the possibilities of comparisons between birth cohorts and examination years. The H70-studies have followed a strict protocol with validated methods over time. The PI of the study (last author IS) took part in the data collection already in 1984, and was trained by the researchers who conducted the first studies in the 1970s. He has supervised and trained research staff in H70 studies in subsequent examinations, including the examination of birth cohort 1944.

A fourth challenge is that examinations were performed by psychiatric nurses, medical doctors or psychiatrists which may have affected rating consistency. We have previously reported good inter-rater reliability between nurses and psychiatrists, e.g. regarding dementia symptoms [83].

A fifth challenge is to keep diagnoses of diseases consistent and at the same time up to date. Following data collection, which comprises extensive personal examinations using structured interviews, all diagnoses were made by an expert team of experienced psychiatrists and medical doctors. In addition, psychiatric diagnoses were further established by using algorithms including individual symptoms and signs according to criteria in the Diagnostic and Statistical Manual of Mental Disorders (DSM). This was made possible as symptom ratings, rather than questions about diagnoses, have been the core part of the studies since 1971. This enables comparisons between studies irrespective of modifications in criteria and guidelines.

## Conclusion

The research gained from the Gothenburg H70 Birth Cohort Studies has clinical relevance in relation to prevention, early diagnosis, clinical course, experience of illness, understanding pathogenesis and prognosis. Results will increase our understanding of ageing and inform service development, which may lead to enhanced quality of care for older persons.

## Electronic supplementary material

Below is the link to the electronic supplementary material.

Supplementary material 1 (DOCX 41 kb)

Supplementary material 2 (DOCX 41 kb)

Supplementary material 3 (DOCX 41 kb)

Supplementary material 4 (DOCX 40 kb)

Supplementary material 5 (DOCX 41 kb)

For a full list of abbreviations, see Supplementary 6. (DOCX 43 kb)
