# Supplementary material for: The Gothenburg H70 Birth cohort study 2014–16: design, methods and study population
Source: Eur J Epidemiol. 2018 Nov 13;34(2):191–209. doi: 10.1007/s10654-018-0459-8 (PMC6373310; doi:10.1007/s10654-018-0459-8)
Supplement: Supplementary file 1 — Supplementary material 1 (DOCX 41 kb) [file 10654_2018_459_MOESM1_ESM.docx]

**SUPPLEMENTARY 1**

**The assay/reagent and manufacturer for each blood test measure**

**Analytical instrument: ADVIA 2120i Siemens Medical Diagnostics AB**

B-Hemoglobin B-Hb Assay: Advia™ 2120 CN-FREE HGB

B-TPK (ADVIA2120i) Trombocytes, Assay: Advia™ 2120 RBC/PLT.

**Analytical instrument: Cobas® 8000 modular analyzer (Roche Diagnostica Scandinavia AB).** The reagens has CE labelling according to the IVD directive.

P-Homocystein. Assay: HCYS

Alanin aminotranferas (ALAT) in serum: Assay: ALTPM

Aspartat aminotransferas (ASAT) in serum: Assay: ASTPM

Creatinine. Assay; CREP2

Glucose: reagens: GLUC3

Cholesterol assay: CHOL2

Assay: HDL- C Gen. 3.

Assay: LDL_C plus 2nd generation.

Triglycerider. Assay: TRIGL

S-T4 fritt (Cobas e). Reagens: FT4 II Elecsys

TSH: reagens: TSH Elecsys

**Analytical instrument: STA-R Evolution Diagnostica Stago, Asnieres France**

Protrombinkomplex, PK(INR) Reagens: STA-SPA+

**Coagulation instrument**: Sysmex CS-5100 / Siemens Healthcare Diagnostics AB / Upplands Väsby
