# Supplementary material for: The Gothenburg H70 Birth cohort study 2014–16: design, methods and study population
Source: Eur J Epidemiol. 2018 Nov 13;34(2):191–209. doi: 10.1007/s10654-018-0459-8 (PMC6373310; doi:10.1007/s10654-018-0459-8)
Supplement: Supplementary file 2 — Supplementary material 2 (DOCX 41 kb) [file 10654_2018_459_MOESM2_ESM.docx]

**SUPPLEMENTARY 2**

**Extended audiological examination**

A subsample of 305 participants, born on dates ending with 2 and 5 of each month, were invited for an extended audiological examination conducted at the Audiology Unit, Sahlgrenska Academy. Of those invited, 251 participants (113 men, 138 women; response rate 82.3 %) underwent the examination with total duration of approximately 90 minutes. Non-participants reported several reasons for declining (e.g. experienced no need for additional testing beyond the audiological examination described above, ongoing contact with an audiological unit, unwilling to participate in any or further additional examinations). All examinations were performed by qualified audiologists, with soundproof conditions, adhering to ISO 8253-1 [79]. Prior to testing, otoscopy and a brief medical interview was conducted. Those with occluding ear wax (n=3) were referred for wax removal and retested at a later point. The extensive test battery consisted of widely used psycho-acoustic and physiologic methods aiming to investigate the auditory function from ear to brain in detail. Hearing sensitivity was measured with pure-tone audiometry. Air and bone conduction thresholds were determined according to standardized methodology [79] using an Interacoustics Equinox AC33 audiometer and Telephonics TDH-39 headphones. Speech recognition scores in noise (S/N+4) were obtained using phonemically balanced lists of 50 monosyllabic words [80]. To test the central auditory function, the Dichotic digits test with single digit pairs was performed, according to a method described previously [81]. Both free report and direct report scores were recorded. Middle ear function was assessed with clinical tympanometry, performed with an Interacoustics Titan. The function of outer hair cells in the cochlea was investigated through distortion product otoacoustic emissions (DPOAE) with Otodynamics Echoport ILO292-II equipment. Responses were recorded at 6 test frequencies (1-6 kHz) using a 70/70 dB SPL stimulus. Auditory nerve function was assessed with auditory brain response (ABR) using an Interacoustics Eclipse (EP25) with EAR insert earphones. Both diagnostic and threshold ABRs were recorded with a click stimulus. Participants who were identified with hearing loss were offered hearing aid consultations, and referred to the audiology clinic when appropriate.
