# Supplementary material for: The Gothenburg H70 Birth cohort study 2014–16: design, methods and study population
Source: Eur J Epidemiol. 2018 Nov 13;34(2):191–209. doi: 10.1007/s10654-018-0459-8 (PMC6373310; doi:10.1007/s10654-018-0459-8)
Supplement: Supplementary file 3 — Supplementary material 3 (DOCX 41 kb) [file 10654_2018_459_MOESM3_ESM.docx]

**SUPPLEMENTARY 3**

**Extended ophthalmic examination**

A subsample of 631 participants, born on days ending with 0, 2, 5 and 8 (born January-April), and 5 and 8 (born May-December), were invited for an ophthalmic examination conducted at Sahlgrenska University Hospital, Mölndal. Of those invited, 561 participants (266 men, 295 women; response rate 88.9 %) underwent the examination with a total duration of approximately 90 minutes. Non-participants reported several reasons for declining (e.g. lack of time, ongoing contact with an ophthalmic unit). The ophthalmic examination included tests of presenting visual acuity and best-corrected visual acuity at far distance as well as contrast sensitivity. Visual fields were examined with frequency doubling test (FDT) as a screening tool and those showing visual field defects were tested using Humphrey perimeter. To evaluate anterior and posterior segments of the eye, images of the lens and fundus were taken after instillation of mydriatic drops and optical coherence tomography was performed. The examination protocol was designed to primarily diagnose cataract, glaucoma, age-related macular degeneration (AMD) and diabetic retinopathy. After the examination, participants received information about eyesight, if glasses/lenses were in need of adjustment and some general information about the anatomy of the eye. All ophthalmic data and imaging of the eyes were evaluated by an ophthalmologist. Participants with newly detected abnormalities were called for further examination and treatment by an ophthalmologist.
