# Supplementary material for: The Gothenburg H70 Birth cohort study 2014–16: design, methods and study population
Source: Eur J Epidemiol. 2018 Nov 13;34(2):191–209. doi: 10.1007/s10654-018-0459-8 (PMC6373310; doi:10.1007/s10654-018-0459-8)
Supplement: Supplementary file 4 — Supplementary material 4 (DOCX 40 kb) [file 10654_2018_459_MOESM4_ESM.docx]

**SUPPLEMENTARY 4**

**Qualitative studies**

Purposive sub-samples of study participants also took part in qualitative studies (focus groups or individual interviews). In the first study (n=38), the experience of participating in the H70 study conducted in 2014-16 was discussed in nine focus groups. In the second study (n=16), the experience of having depression in late life was discussed in four focus groups. In the third study (n=15), individual interviews were performed comprising questions about meals and meal planning from a health literacy perspective.
