# Supplementary material for: The Gothenburg H70 Birth cohort study 2014–16: design, methods and study population
Source: Eur J Epidemiol. 2018 Nov 13;34(2):191–209. doi: 10.1007/s10654-018-0459-8 (PMC6373310; doi:10.1007/s10654-018-0459-8)
Supplement: Supplementary file 5 — Supplementary material 5 (DOCX 41 kb) [file 10654_2018_459_MOESM5_ESM.docx]

**SUPPLEMENTARY 5**

**Registry data**

The H70 study is permitted to be linked to a large number of databases from different state agencies in Sweden (e.g. including data regarding participants’ birth records, visits to early child care, schooling, drafting records for 18-year-old men). These include Statistics Sweden, the National Board of Health and Welfare (the National Patient Register, the Swedish Cancer Registry, the Cause of Death Register, the Swedish Prescribed Drug Register, the Register for Care and Social Services), the Swedish Tax Agency, Region Västra Götaland (information on primary care, hospital care and specialist care within and outside the Region of Västra Götaland from the VEGA database), the Centre of Registers Västra Götaland (Regional Quality Registry for Primary Care - QRegPV), Swedish National Quality Registries (national registers for e.g. stroke, dementia, diabetes, fractures, cardiovascular diseases) and for men, the Swedish Defence Recruitment Agency (The Swedish Military Service Conscription Register which includes conscription data on e.g. cognitive performance, blood pressure, length, and weight at age 18 years).
