# Supplementary material for: The Gothenburg H70 Birth cohort study 2014–16: design, methods and study population
Source: Eur J Epidemiol. 2018 Nov 13;34(2):191–209. doi: 10.1007/s10654-018-0459-8 (PMC6373310; doi:10.1007/s10654-018-0459-8)
Supplement: Supplementary file 6 — For a full list of abbreviations, see Supplementary 6. (DOCX 43 kb) [file 10654_2018_459_MOESM6_ESM.docx]

**SUPPLEMENTARY 6**

**List of abbreviations**

MRI: magnetic resonance imaging; Hb: hemoglobin; TSH: thyroid stimulating hormone; T4: Thyroxine (thyroid hormone); AST: aspartate aminotransferase; ALT: alanine aminotransferase; HDL: High-density lipoprotein; LDL: Low-density lipoprotein; DNA: Deoxyribonucleic acid; UK: United Kingdom; SNPs: Additional single nucleotide polymorphisms; CPRS: Comprehensive Psychopathological Rating Scale; MINI: the Mini-International Neuropsychiatric Interview; MADRS: The Montgomery-Åsberg Depression Rating Scale; BSA: The Brief Scale for Anxiety; GAF: Global Assessment of Functioning; ADHD: Attention deficit hyperactivity disorder; DSM: the Diagnostic and Statistical Manual of Mental Disorders; PSS-14; The Perceived Stress Scale comprising 14 items; MMSE: the Mini-Mental State Examination; GBS: the Gottfries Bråne Steen-scale; CDR: the Clinical Dementia Rating scale; ADAS-COG: Alzheimer’s Disease Assessment Scale-Cognitive; MIR: Memory in Reality; BUS II: Supra-span memory test; SRB 2: Figure Logic test; COWA-FAS: Controlled Oral Word Association – FAS; TIA: transient ischemic attack; SF-36: The Short-Form Health Survey comprising 36 questions; ATC: Anatomical Therapeutic Chemical; WHO: World Health Organization; AUDIT: The Alcohol Use Disorders Identification Test; DUDIT: The Drug Use Disorders Identification Test; ECG: Electrocardiography; FVC: forced volume capacity; FEV1: forced expiratory volume in 1 second; PEF: peak expiratory flow; FEF: forced expiratory flow; MMEF: maximal (mid-) expiratory flow; SVC: slow vital capacity; FET: forced expiratory time; VFQ-25: The Visual Function Questionnaire comprising 25 questions; ADL: activities of daily living; IADL: Instrumental Activities of Daily Living Scale; IPAQ: The International Physical Activity status Questionnaire; ICECAP-O: The ICEpop CAPability measure for Older people; SOC: sense of coherence; CMPS: The Cesarec Marke Personality Scheme; EPI: The Eysenck Personality Inventory; NEO-FFI-3: The NEO Five Factor Inventory; PN-SRI: The Positive-Negative Sex-Role Inventory; CIRS-G: the Cumulative Illness Rating Scale for Geriatrics; DXA: dual energy X-ray absorptiometry; BIS: Bioelectrical impedance spectroscopy; CT: computed tomography; MRI: magnetic resonance imaging; LP: lumbar puncture; IQCODE: the Informant Questionnaire on Cognitive Decline in the Elderly; DH: the diet history method; FLAIR: fluid attenuation inversion recovery; t2w: T2 weighted images; DTI: diffusion tensor imaging; fMRI: Functional magnetic resonance imaging; CSF: Cerebrospinal fluid; RCF: Relative Centrifugal Force; Aβ42: Amyloid- β42; ELISA: enzyme-linked immunosorbent assay; β-amyloid: amyloid beta; ISO: International Organization for Standardization; DPOAE: distortion product otoacoustic emissions; ABR: auditory brain response; FDT: frequency doubling test; AMD: age-related macular degeneration; VEGA: Västra Götaland’s care database; PI: principal investigator; EDI: Questionnaire regarding body image and emotions attitudes towards eating; GDPR: The General Data Protection Regulation (EU) 2016/679; FAIR: The FAIR Data Principles (Findability, Accessibility, Interoperability, Reusability).
